# Histological validation of high-resolution DTI in human post mortem tissue

**DOI:** 10.3389/fnana.2015.00098

**Published:** 2015-07-23

**Authors:** Arne Seehaus, Alard Roebroeck, Matteo Bastiani, Lúcia Fonseca, Hansjürgen Bratzke, Nicolás Lori, Anna Vilanova, Rainer Goebel, Ralf Galuske

**Affiliations:** ^1^Faculty of Psychology and Neuroscience, Maastricht UniversityMaastricht, Netherlands; ^2^Systems Neurophysiology, Technische Universität DarmstadtDarmstadt, Germany; ^3^Jülich Research Centre, Institute of Neuroscience and Medicine (INM-4)Jülich, Germany; ^4^Department of Biomedical Engineering, Eindhoven University of TechnologyEindhoven, Netherlands; ^5^Institute of Forensic Medicine, Goethe-UniversityFrankfurt, Germany; ^6^Visual Neuroscience Laboratory, Institute for Biomedical Imaging and Life Sciences (IBILI), Faculty of Medicine, University of CoimbraCoimbra, Portugal

**Keywords:** diffusion tensor imaging, histological validation, fiber orientations, gray matter, diffusion microstructure

## Abstract

Diffusion tensor imaging (DTI) is amongst the simplest mathematical models available for diffusion magnetic resonance imaging, yet still by far the most used one. Despite the success of DTI as an imaging tool for white matter fibers, its anatomical underpinnings on a microstructural basis remain unclear. In this study, we used 65 myelin-stained sections of human premotor cortex to validate modeled fiber orientations and oft used microstructure-sensitive scalar measures of DTI on the level of individual voxels. We performed this validation on high spatial resolution diffusion MRI acquisitions investigating both white and gray matter. We found a very good agreement between DTI and myelin orientations with the majority of voxels showing angular differences less than 10°. The agreement was strongest in white matter, particularly in unidirectional fiber pathways. In gray matter, the agreement was good in the deeper layers highlighting radial fiber directions even at lower fractional anisotropy (FA) compared to white matter. This result has potentially important implications for tractography algorithms applied to high resolution diffusion MRI data if the aim is to move across the gray/white matter boundary. We found strong relationships between myelin microstructure and DTI-based microstructure-sensitive measures. High FA values were linked to high myelin density and a sharply tuned histological orientation profile. Conversely, high values of mean diffusivity (MD) were linked to bimodal or diffuse orientation distributions and low myelin density. At high spatial resolution, DTI-based measures can be highly sensitive to white and gray matter microstructure despite being relatively unspecific to concrete microarchitectural aspects.

## Introduction

Diffusion Magnetic Resonance Imaging (dMRI) is a widely used MRI technique in clinical as well as basic neuroscience applications to reveal neuronal fiber structures non-invasively in the living brain. A dMRI acquisition provides information about water diffusion in several directions in space. Usually this information is integrated in a mathematical model to give a unified description of the diffusion in one voxel. One of the mathematically simplest and yet the most widely used of these models is diffusion tensor imaging (DTI) (Basser et al., 1994; see Mori and Zhang, 2006 for an overview). In a diffusion tensor, diffusion is characterized as a Gaussian function with 3 orthogonal diffusion axes along with their characteristic lengths. Different characteristics of diffusion tensors are described by scalar measures such as mean diffusivity (MD; the average axis length) or fractional anisotropy (FA; the normalized variance of diffusion over the axes). MD, in particular, has been successfully used in a range of clinical diagnostic cases (Sundgren et al., 2004). FA is widely used in neuroscience and pre-clinical investigations as a sensitive marker of white matter microstructure (Kanaan et al., 2005; Medina and Gaviria, 2008; Richardson, 2010), but its microstructural basis is still under debate (Assaf and Basser, 2005; Assaf et al., 2013; Jones et al., 2013). Furthermore, DTI can be used for tractography, which is the algorithmic concatenation of neighboring tensors, yielding inferred fiber pathways. The latter is an important tool in *in-vivo* brain connectivity research (Mori et al., 1999; Catani and Thiebaut de Schotten, 2008), including the relatively young field of human connectomics (Sporns et al., 2005; Hagmann et al., 2008).

Despite its extensive usage, DTI has the drawback in tractography of being a rather unspecific unidirectional model. Its orientation estimation works very well in areas characterized by prominent fiber pathways following one direction, giving rise to a unimodal water diffusivity profile. This may often be the case, especially in white matter. However, when several different diffusion directions are present in one voxel, the directionality information of the estimated diffusion tensor is imprecise at best or even systematically biased (Wedeen et al., 2008; Jones, 2010). This is particularly the case in gray matter where neurites more frequently run in at least two orthogonal or non-orthogonal directions. But also in white matter this is a known problem, recently estimated to occur in up to 90% of all voxels (Jeurissen et al., 2013), when fiber tracts cross or touch each other, converge or diverge (Roebroeck et al., 2008).

In order to approach these problems and to gain a better understanding of the exact nature of the dMRI signal and its modeling on the anatomical level, it is very important to conduct validation studies, which compare dMRI data to other sources of information. This information can for example come from phantoms (Pullens et al., 2010), MRI contrast agents (Lin et al., 2001), anatomical atlases (Catani et al., 2002), or a direct comparison of dMRI findings to the actual histological situation in post-mortem tissue (Leergaard et al., 2010; Seehaus et al., 2013). The latter approach is particularly promising for two reasons: First, it allows for a direct comparison of findings gained in both modalities in the very same tissue. Second, it allows for the comparison of different anatomical structures to the dMRI-findings since histological stains can be varied in order to visualize different architectural aspects, such as cell-bodies, dendrites, or myelin.

In two recent studies, Budde and colleagues propose a methodological approach for the validation of DTI with histology (Budde and Frank, 2012; Budde and Annese, 2013). Using structure tensors to detect local image orientation, they extracted microanatomical orientations from digitized stained tissue sections. Pooling this information over image compartments that correspond to the voxel size in a hypothetical imaging experiment, they derived fiber orientation distributions (FODs) which structurally closely resemble real dMRI data. They illustrate their method on a range of different stains in rat and human brain. They also report promising results of a preliminary comparison to actual DTI data where histological anisotropy values were averaged over selected ROIs in the rat brain and correlated with FA averages of the same ROIs.

In our study, we apply this analytical approach to a series of 65 myelin-stained human brain sections in order to validate the orientational and microstructure information obtained from DTI data acquired on the same tissue probe. We extend previous studies by providing for the first time a direct voxel-based comparison of DTI and myeloarchitecture over a relatively large volume of human tissue focusing both on white matter and gray matter. In doing so, our main research questions were: First, how do the orientations of diffusion tensors match those of the underlying fiber material in the presence of both unidirectional and crossing pathways? This question is particularly important in the light of DTI tractography where fiber pathways are modeled by concatenating diffusion tensors along their primary orientations. Second, how do the most important scalar indices of diffusion tensors, FA, MD, and radial diffusivity (RD), relate to myeloarchitecture? Third, how does the microstructural environment, and its quantification by dMRI, change in gray matter? So far, diffusion imaging has mostly been used in white matter, however, there is an increasing number of recent studies investigating human gray matter with high resolution dMRI (Kleinnijenhuis et al., 2013; Leuze et al., 2014).

## Materials and methods

### Tissue acquisition

This study was performed on a block of cortical tissue (38.9 × 36.3 × 23.8 mm) which comprised parts of primary motor and medial and lateral premotor cortex. The tissue was obtained 6 h post mortem from the left hemisphere of a female subject, aged 38, without known neurological or psychiatric disorders. All procedures were approved by the ethical committee of the University Hospital Frankfurt/M. The tissue was prepared and fixed for 48 h using a solution containing 2.6% paraformaldehyde, 0.8% iodoacetic acid, 0.8% sodium periodate, and 0.1 M D-L-lysine in 0.1 M phosphate buffer at pH 7.4 at 4°C. For long term storage, it was then transferred to a 2% paraformaldehyde solution in 0.1 M phosphate buffer at pH 7.4. MR scans were performed after about 1 year of fixation. The tissue was scanned in a cylindrical container immersed in the paraformaldehyde solution in order to assure long-term preservation for later histological processing.

### Diffusion MRI

Measurements were performed on a small-bore 9.4T system equipped with a 12 cm ID, 600 mT/m, 100 μs rise time gradient coil and interfaced to a Siemens console. A 7 cm loop-coil was used for RF transmission and signal reception. A 2D spin-echo sequence was modified to include a diffusion preparation module. The measurement parameters included: FOV 54 × 54 mm^2^, matrix 160 × 160, 97 contiguous 340 μm slices (achieving isotropic resolution of 340 μm), *TR* = 10,000 ms, *TE* = 45 ms, Δ = 22.5 ms, δ = 3 ms, flip angle = 90°, 4 averages, *b* = 3000 s^*^mm^−2^, 60 diffusion encoding directions (obtained by an electrostatic repulsion algorithm on the whole sphere) and six *b* = 0 acquisitions. The temperature in the scanner was raised to 30°C using an in-bore animal warming system and constantly monitored with a temperature probe.

The signal-to-noise ratio of the acquired data was estimated in the *b* = 0 acquisitions as the signal within the tissue divided by the standard deviation in an image corner free of signal and evaluated to 51. Diffusion data were preprocessed in order to correct for image shift and geometric distortions arising from eddy currents induced by diffusion gradients using the FMRIB's Diffusion Toolbox available in FSL (Jenkinson et al., 2012). The estimated transformation matrices were used to transform the diffusion gradient directions accordingly (Leemans and Jones, 2009). Manual segmentation of the averaged non diffusion-weighted (i.e., pure T2-weighted) volumes was performed to obtain white and gray matter masks. Diffusion tensors (DTs) were fitted to the acquired data by linear regression using a least square minimization approach. For the diffusion tensors, FA, MD, and RD were determined.

### Histological processing

After the MR scan, the block was cut into two halves, with the cutting plane approximating the xy-plane of the MR space. This was possible due to orientation marks on the container the tissue was scanned in, as well as photographic documentation of the tissue positioning within that container. The anterior part was sectioned at a slice thickness of 60 μm using a microtome (Reichert-Jung, Supercut 2050) with a freezing stage (Leica, Frigomobil). To facilitate orientation within the tissue material later on, blockface photos were taken of every second section (Choe et al., 2011). This resulted in 343 coronal sections, from which every 5th one was stained for myelin using the Gallyas method (Gallyas, 1979; see Supplementary Material for a detailed staining protocol). In this manner, we obtained 69 stained sections. Of those, 4 had to be discarded due to damage during the staining procedure, leaving 65 sections for microscopical analysis.

Furthermore, every 20th section was Nissl-stained for the identification of cortical layers and area classification. In accordance with the atlas of Economo and Koskinas (1925), the analysis of the Nissl-stained sections confirmed our macroscopic area classification during tissue acquisition. Myelin stained sections were digitized using a microscope system consisting of microscope (Zeiss, AxioImager Z1), motorized stage (Märzhäuser), and camera (Zeiss, Axiocam HRm). Digital images were obtained with a 5x magnification objective as series of 1388 × 1040 px sized tiles, which were automatically merged using the built-in stitching algorithm (Zeiss, MosaiX). The resulting images were 8 bit grayscale, with a pixel resolution of 1.3 μm^2^.

### Histological orientation analysis

The aim of the histological analysis was to obtain fiber orientation distributions from image compartments equaling the DTI voxel size. This was achieved in a stepwise process:

#### Image preprocessing

Histological procedures such as the one used here usually stress the tissue both physically and chemically, resulting in tissue damage such as tears and ripples. Particularly around vessels, the tissue sections develop holes during the process of exsiccation. As a first step, these damaged parts had to be discarded, which was done by means of manual segmentation, using standard image processing software (Adobe Photoshop CS2). Additionally, we discarded parts of the tissue which did not contain stained fibers, mostly situated in cortical layers I and II.

#### Structure tensor calculation

There are several different approaches to calculate orientedness in digital images, including integral transformations like Fourier or Wavelet decomposition (Kemao and Asundi, 2002; Lefebvre et al., 2011), oriented filters (Michelet et al., 2007), or template matching (Bartsch et al., 2012). Because of its recent success, we chose the structure tensor approach (Bigün and Granlund, 1987), which is based on local gray level gradients.

The following algorithm was conducted on each histological section image: First, the image gradient (*f*_*x*_, *f*_*y*_) was calculated by convolution of the image matrix with the partial derivatives *G*_*x*_, *G*_*y*_ of a rotationally symmetric Gaussian kernel G (size 9 pixels, σ = 2).

From these gradient images, the structure tensors (STs) were acquired as

$$\begin{array}{r} J=\left[\begin{array}{cc} f_{xx} & f_{xy}\\ f_{xy} & f_{yy} \end{array}\right] \end{array}$$

where *f*_*xx*_ is the pointwise product of *f*_*x*_ with itself, smoothed by convolution with G (analogously for *f*_*xy*_, *f*_*yy*_).

Subsequently, in each pixel the eigenvalues λ_1_, λ_2_ (where we will assume λ_1_ ≥ λ_2_) and eigenvectors v_1_, v_2_ of the STs were calculated. The primary image orientation can be obtained from the larger eigenvector, hence the planar angle:

$$\begin{array}{r} \varphi_{ST}=arctan\left(\frac{v_{1,y}}{v_{1,x}}\right) \end{array}$$

of v_1_ describes the sought-for orientation in a pixel.

#### Creating a histological voxel space

In order to make histological and DTI data comparable, the histological data had to be converted from their original spatial resolution to the DTI voxel resolution. This was done by partitioning the histological sections into subimages measuring 340 × 340 μm and using different operations to downsample the histological values determined in each pixel to subimage or “voxel” values. The gray level of a voxel was obtained by taking the average over the subimage pixel values. Via the expression: 1—gray level, it served as a measure of staining intensity in the statistical analysis. For calculation of voxel orientations, simple averaging was not applicable due to the periodicity of circular data. Instead, for each voxel a 180 bin (= 1° angular resolution) histogram of all pixel orientations was created, normalized (division by the subimage pixel number) and denoised by convolution with a Gaussian window of 23° full width at half maximum (FWHM). The smoothed histograms were then fitted with mixtures of *von Mises* distributions, which closely resemble wrapped normal distributions (Lee, 2010). The fitting of three *von Mises* components on each smoothed histogram h was conducted using the following algorithm:

1. Find the maximum (*x*_*max*_, *y*_*max*_) of *h*. Take it as center θ and amplitude d of a *von Mises* component $\frac{d}{f_{\theta ,\kappa }\left(x_{max}\right)}f_{\theta ,\kappa }$ with f the *von Mises* probability density function. Due to the axial nature of the data [*f*_(0°)_ = *f*_(180°)_], f was defined on the half instead of the full circle.
2. Determine the dispersion parameter κ of that component by minimizing the square error in a local neighborhood around θ, i.e., solve the optimization problem
  $$\begin{array}{r} {min}_{\kappa }\overset{\theta \;+\;42}{\underset{x\;=\;\theta \;-42}{\sum }}{\left(h\left(x\right)-\frac{d}{f_{\theta ,\kappa }\left(x_{max}\right)}f_{\theta ,\kappa }\left(x\right)\right)}^{2} \end{array}$$
3. Replace *h* with the residual $h-\frac{d}{f_{\theta ,\kappa }\left(x_{max}\right)}f_{\theta ,\kappa }$, repeat.

**Figure 1:**
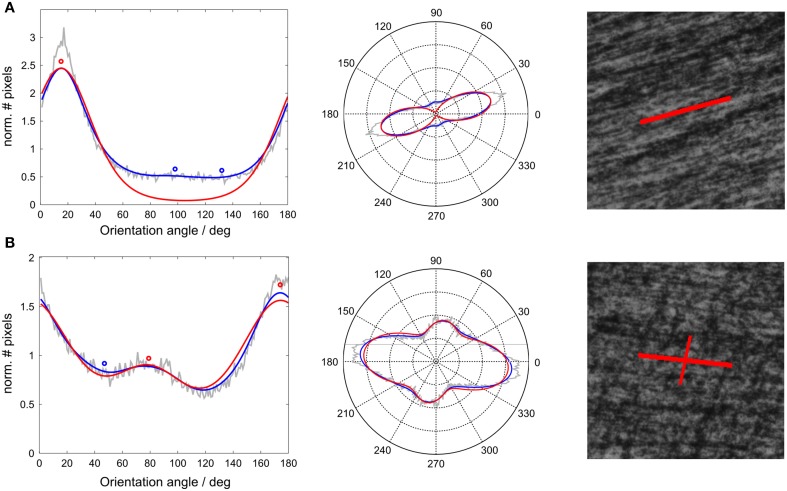
**Histological fiber orientation distribution with fitted ***von Mises*** mixture for two exemplary voxels**. Left: Normalized histograms of structure tensor orientations of all pixels within a voxel (gray line), smoothed with a Gaussian window (blue line) and fitted using a mixture of three *von Mises* probability density functions (red line). The central values of the *von Mises* components were the resulting histological orientations in a voxel (circles). Middle: Same histograms as polar plots. Right: High amplitude voxel orientations (corresponding to red circles) visualized on corresponding histological tile. **(A)** Example of 1-orientation voxel, **(B)** 2-orientation voxel.

The algorithm was implemented in Matlab, 2012, making use of a freely available toolbox for circular statistics (Berens, 2009). In summary, this algorithm returned the values of theta, kappa, and amplitude for each of three *von Mises* components. The theta values should indicate in descending order the directions of the three most prominent fiber directions in a histological voxel. The first of these values was considered as the primary orientation Φ_*ST*_ of a voxel and was used to determine the orientation differences to DTI-results. In theory, this method was capable of accurately identifying three directions within an image. In practice however, only the primary and the secondary orientations reliably represented fiber directions (Figure 1). From the kappa value, which indicates the “narrowness” of a component, its *width* was inferred via *1*-κ∕κ_*max*_, with κ_max_ the maximum over all analyzed voxels. Both amplitude and width of the primary component are measures of how peaked, i.e., anisotropic, the fiber orientation distribution is. Therefore, we expected these values to be correlated to FA as well as the orientation match with DTI.

### Alignment

The MRI and the histological volume were coregistered using a 3D affine transformation, a combination of rotations, translations, and scales. Since the tissue was sectioned precisely along the xy-plane of the MRI scan, a rotation in the xz and yz-planes was unnecessary. Furthermore, as the histological volume was not 3D but a series of 2D sections, the scaling and translation along the z-axis was replaced by a (manually conducted) assignment between histological sections and MRI z-slices. The MRI voxel resolution was 340 μm in each direction while neighboring sections were 300 μm apart from each other (60 μm thickness ^*^ every 5th section stained), so this was approximately a 1:1 assignment with each 10th z-slice mapped onto two consecutive histological sections. For the resulting section-slice pairs, the remaining operations were one rotation (xy-plane), two translations (x and y-direction) and one scaling (xy-direction—exploration revealed that separate scaling in x and y-direction was unnecessary). These operations were performed using a custom-made graphical user interface in Matlab, 2012a where the section images could be gradually moved onto the respective slices. In this way, a transformation, represented as a 3 × 3 matrix in homogeneous coordinates, was determined for each section-slice pair, translating histological into MRI coordinates and vice versa.

### Diffusion tensor orientations

In order to obtain planar orientation angles from the diffusion tensors, in each voxel the eigenvector *w*_*max*_ corresponding to the largest eigenvalue was projected into the sectioning plane (since the sectioning plane was the xy-plane of the MRI volume, this was achieved by discarding the z-coordinate). From this projection, the orientation angle was obtained as

$$\begin{array}{r} \Phi_{DT}=arctan\left(\frac{w_{max,y}}{w_{max,x}}\right) \end{array}$$

Of course, the projection is shorter the more perpendicularly *w*_*max*_ is oriented toward the xy-plane, resulting in information loss. The same holds for the histological data, where orthogonally cut fibers appear as dots. To alleviate this problem, only voxels with an out-of-plane angle of maximally 45° were used for further analyses.

### Statistical evaluation

#### Orientation differences

The difference between DT- and ST-based voxel orientations in axis angles was calculated as

$$\begin{array}{r} d\left(\Phi_{DT},\Phi_{ST}\right)=min\left(\left|\Phi_{DT}-\Phi_{ST}|,180-|\Phi_{DT}-\Phi_{ST}\right|\right) \end{array}$$

The central tendency of these differences across voxels was measured in terms of arithmetic mean and median. This was done for the entire volume as well as individually for gray/white matter and for different ranges of FA.

#### Correlations and regression

Correlations across voxels were calculated between a range of values derived from DTI (FA, MD, RD), histology (staining intensity, amplitude and width parameters of the *von Mises* fit), or both combined (orientation difference).

Additionally, multiple linear regression was used to predict FA, MD, and RD from the histology-based variables. The overall amount of predictability is reported as percentage of explained variance (R^2^).

As the intensity of the myelin stain varied between the sections due to technical reasons, calculation of correlations and regressions over the entire volume would not have led to reliable results. Instead, these calculations were performed for each section individually. We report the mean of the section results, weighted with the number of voxels in each section.

## Results

### Sample sizes

A total of 65 histological sections were mapped onto 60 volume slices. In voxel space (Cartesian coordinate system with 340 μm unit length), this provided a sample of *N*_*Pair*_ = 221,681 voxels for which both a diffusion tensor and histological structure tensors were available. 6.13% of all voxels were discarded due to tissue damage and 41.11% for not including stained material. Of the remaining voxels, 53.68% were within the threshold for the out-of-plane angle (see Diffusion Tensor Orientations), leading to a subset of *N* = 62,782 voxels as the data set for subsequent analyses. Out of these, 27.6% of voxels were located within gray matter, and 72.4% within white matter.

### Orientation differences

**Figure 2:**
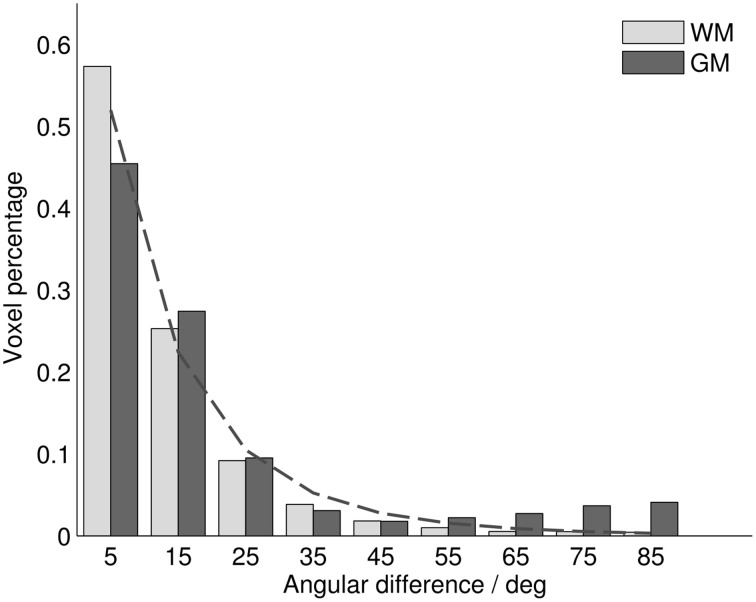
**Histogram of angular differences between MRI_DT_ and Hist_ST_ orientations over all analyzed voxels for white and gray matter**. Dashed line: Histogram fit with generalized Pareto distribution.

**Figure 3:**
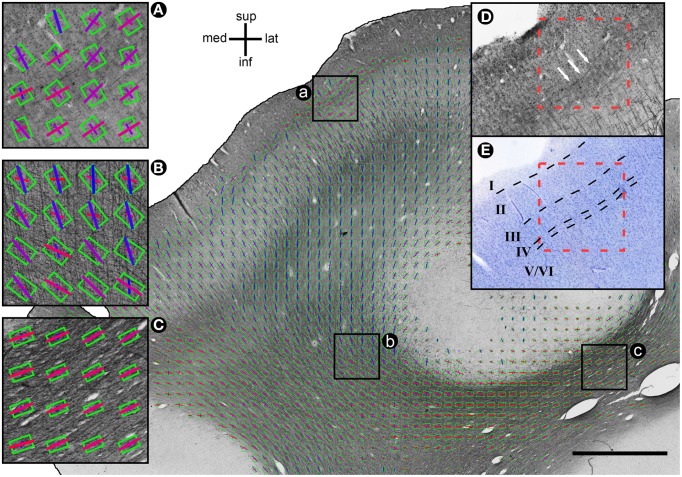
**Exemplary histological section with MRI_DT_ and Hist_ST_ orientations**. Diffusion tensors are coded by oriented green rectangles, where rectangle aspect ratio indicates fractional anisotropy (more elongated = higher FA). Hist_ST_ orientations are coded by bars. The color of the bars indicates the respective orientation (lateral-medial = red; inferior-superior = blue). Size of DT rectangles and ST bars is proportional to DT projection length. Scale bar: 3 mm. **(A)** Voxels in which myelin orientations are parallel to the cortical surface, DT orientations orthogonal. **(B)** Fiber crossing. FA is low and the DT orientation is in between the primary and secondary histological orientation. **(C)** 1-orientation fiber pathway. FA is high, the secondary histological orientations are very small, and there is a good match of primary histological and DT orientation. **(D)** Area (a) without DT and ST information. Arrows indicate the tangential fibers determining the ST orientations. **(E)** Classification of cortical layers in an area corresponding to area (a) in a neighboring Nissl-stained section.

Throughout all analyzed voxels, the average difference between MRI diffusion tensor (MRI_DT_) and histology structure tensor (Hist_ST_) orientations was 14.25°, with a median of 9.01°. Differences were generally larger in gray matter (mean 19.51°, median 11.25°) than in white matter (12.25°, 8.34°). In both tissue types, the distribution of orientation differences resembled a power law, with most voxels showing differences smaller than 10°, and with a long distribution tail (Figure 2). Mathematically, this was best described with a generalized Pareto distribution (explained variance *R*^2^_WM_ = 0.996, *R*^2^_GM_ = 0.960). This implies that mean orientation differences (being sensitive to large values in the distribution tail) might give an inflated interpretation of central tendency, which is better reflected by the remarkably smaller difference medians. In gray matter, a deviation from this distribution could be observed near 90°, revealing an increased tendency toward orthogonally oriented MRI_DT_ and Hist_ST_ orientations. Most of these voxels were arranged in cortical bands, where stained fibers ran parallel to the cortical surface, while the diffusion tensors fanned out radially from white matter (Figures 3A, 6C).

This phenomenon accounted for most of the difference in orientation match between gray and white matter: When truncated at 50°, the mean difference in white (10.85°) and gray (11.74°) matter was much more similar.

**Figure 4:**
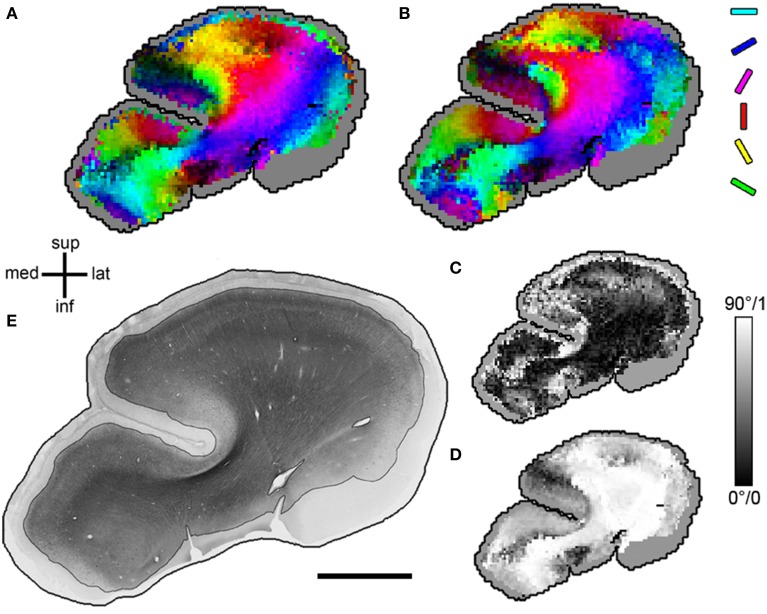
**MRI_DT_ and Hist_ST_ orientations in one exemplary section**. **(A)** Diffusion tensor orientations projected into the sectioning plane. Orientation angle is coded as hue (with the full color spectrum to optimize orientation contrast), projection length as brightness in HSB space. **(B)** Primary structure tensor orientations, same color code as **(A)**. **(C)** Mapping of angular difference between **(A,B)** in axis angles (ranging from 0 to 90°). **(D)** Mapping of projection length of diffusion tensors into the sectioning plane (ranging from 0 to 1). Note that a short projection length is a possible but not the only source of high angular difference in **(C)**. **(E)** Original section with stained area emphasized. In **(A–D)**, the parts without stained fiber material are displayed in gray. Size **(A–D)**, 1 image pixel equals 1 voxel (340 μm); **(E)**, scale bar = 5 mm.

Throughout both white and gray matter, voxels with multiple fiber orientations, due to e.g., crossings, showed remarkable differences between histological and DTI orientations, as shown in Figure 3. In these voxels, the diffusion tensors often represented either a mixture of the existing fiber orientations (Figure 3B for relatively low angle crossings in WM) or a selection of one of the two directions (Figure 3A for the orthogonal crossings in gray matter where DTI picks the radial orientation). Voxels with single fiber orientations (Figure 3C) are well represented by the DTI orientation. Figure 4 shows the spatial distribution of orientation differences for one exemplary section.

#### Relation of FA to angular differences

**Figure 5:**
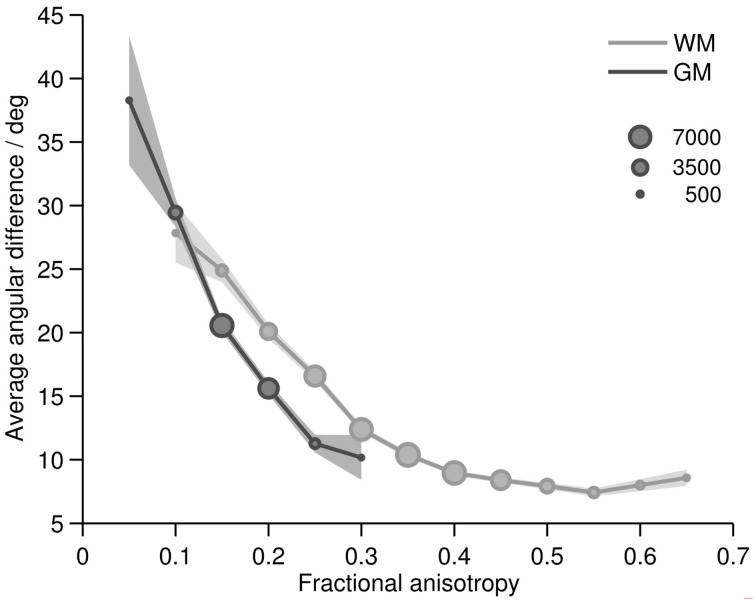
**Average difference between main MRI_DT_ and Hist_ST_ orientations in gray (dark line) and white (light line) matter as a function of FA**. Marker size linearly represents the sizes of the voxel subsamples of the FA bins. FA bins with less than 100 voxels are not displayed. Confidence interval: 1.96* standard error.

Plotting the angular difference over tissue type and FA, we found that, as expected, FA depended largely on the tissue type (WM and GM) and that the angular difference was a function of both tissue type and FA (Figure 5). In white matter, most voxels showed FA values between 0.25 and 0.45. For an FA greater than 0.3, the angular difference was smaller than 11°. For FA values lower than that, the orientation agreement with histology decreased remarkably and monotonically with decreasing FA. In gray matter, mostly FA values between 0.15 and 0.2 were observed. Here the angular difference was between 15 and 21° which is more than the average white matter difference, but less than the white matter difference at this FA level. For FA values lower than 0.15, the orientation match again was very poor, eventually approaching chance level for FA of 0.05.

**Figure 6:**
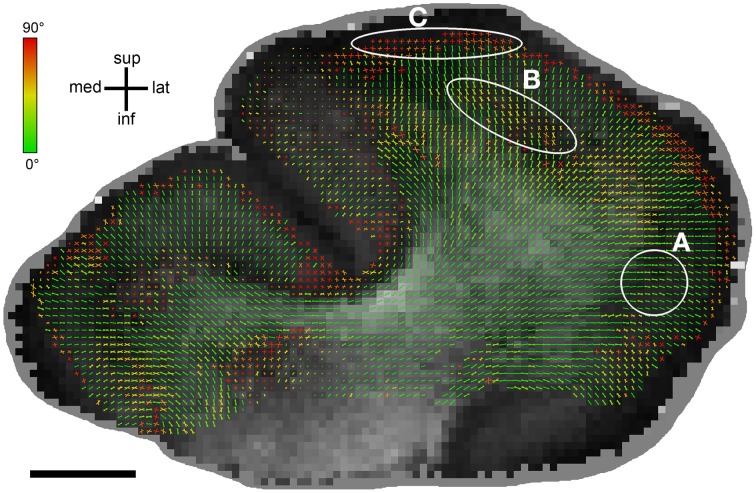
**Differences between MRI_DT_ and Hist_ST_ orientations displayed on an FA map**. MRI_DT_ and Hist_ST_ orientations are plotted in a color range from green (for orientations that perfectly agree) to red (for orientations that differ by 90°). Bar length reflects the projection length of the diffusion tensor in the sectioning plane. There is a generally good histology-DTI agreement for high FA values, especially in white matter. For lower FA, particularly when entering gray matter, there are three cases: **(A)** Good agreement in case of predominant radial orientations, **(B)** Moderate to weak agreement, **(C)** Perpendicular histology-DT orientedness. Here, voxels are located well within gray matter and run in bands parallel to the cortical surface. Scale bar: 5 mm.

The relation between FA and orientation difference was additionally explored visually by plotting histological and DT orientations on FA maps (Figure 6). Overall, it clearly appears that good orientation agreement is achieved where FA is high. However, there are also areas observable where this was not the case, particularly at the transition from white to gray matter. There, FA often decreased significantly (cf. Miller et al., 2011) while the orientation difference remained small.

### Correlation and regression results

Pairwise correlations between different parameters obtained from the MRI and the histological volume were calculated to quantify linear connections between the two data sources, particularly between the scalar measures of DTI and myeloarchitectonic characteristics. An overview of the correlation results is given in Table 1. Fractional anisotropy was strongly correlated to the staining intensity (0.487), the primary and secondary *von Mises* components' amplitudes (0.478, −0.371), and to some degree with the orientation difference d(Φ_DT_, Φ_ST_) (−0.303). The correlation patterns of mean diffusivity and radial diffusivity were similar to that of FA but with opposite signs. Compared to FA, MD, and RD correlated more strongly, but negatively, with staining intensity (−0.542, −0.554) and the amplitude of the secondary orientation (0.397, 0.405), but less strongly with the main orientation amplitude (−0.309, −0.374).

**Table 1:** **Correlation table of histological and dMRI features**.

	**Diff_ST−DT_**	**FA**	**MD**	**RD**	**Stain**	**Ampl_1_**	**Width_1_**	**Ampl_2_**
Diff_ST−DT_	1	−**0.303**	0.293	**0.315**	−0.23	−**0.325**	0.293	0.158
FA	−**0.303**	1	−**0.76**	−**0.854**	**0.487**	**0.478**	−**0.394**	−**0.371**
MD	0.293	−**0.76**	1	**0.983**	−**0.542**	−**0.309**	0.222	**0.397**
RD	**0.315**	−**0.854**	**0.983**	1	−**0.554**	−**0.374**	0.285	**0.405**
Stain	−0.23	**0.487**	−**0.542**	−**0.554**	1	**0.313**	−0.247	−0.285
Ampl_1_	−**0.325**	**0.478**	−**0.309**	−**0.374**	**0.313**	1	−**0.977**	−0.105
Width_1_	0.293	−**0.394**	0.222	0.285	−0.247	−**0.977**	1	−0.086
Ampl_2_	0.158	−**0.371**	**0.397**	**0.405**	−0.285	−0.105	−0.086	1

*Features are: Angular difference between primary Hist_ST_ and MRI_DT_ orientation (Diff_ST−DT_), fractional anisotropy (FA), mean diffusivity (MD), radial diffusivity (RD), staining intensity (1 – voxel gray level), amplitude and width of the primary, amplitude of the secondary Hist_ST_ orientation. Mutual Pearson correlations were calculated separately for each of 65 sections and averaged weighted by the respective voxel counts (standard deviations were always smaller than 0.2). Correlations with an absolute value greater than 0.3 are displayed in bold font*.

Aside from its correlation with FA and MD, the histological FOD as represented by the amplitude of the primary *von Mises* component showed a moderate correlation with the orientation difference (−0.325). This means that the orientations of diffusion tensors and histology tended to be more similar when the FOD was unimodal. The width of the secondary as well as the amplitude and width parameters of the tertiary component showed no strong correlations with DTI-related measures. Predicting FA, MD, and RD from the histological measures (staining intensity, FOD parameters) by means of linear regression, the percentages of explained variance (R^2^) found were 0.472 for FA, 0.449 for MD, and 0.483 for RD.

## Discussion

The aim of this study was to investigate the microstructural basis of diffusion-weighted MRI. We were interested in the quantitative relationship between fiber orientations and scalar characteristics as inferred from a dMRI volume and a histological analysis of the same tissue probe. To this end, fiber orientation distributions from a large number of digitized myelin-stained sections were derived using the structure tensor approach (Schmitt and Birkholz, 2011; Budde and Frank, 2012; Budde and Annese, 2013). While obtained from post-mortem data, we would expect our findings to be generalizable to the *in-vivo* case. Although fixed *ex-vivo* tissue has different MR relaxation parameters (reduced T1 and T2) and reduced water diffusion compared to *in-vivo* tissue, diffusion anisotropy, the crucial marker for neurite orientation and microstructure estimation, remains intact. This means that our correlation, regression and orientation findings should translate well, even if higher absolute values of scalar measures can be expected *in-vivo*.

### Orientations

The angular differences between diffusion tensor and histology-based fiber orientations were taken as an indicator of the goodness of fit of the diffusion tensor as a model of different white matter configurations and gray matter myeloarchitecture.

Our analysis revealed that these differences followed a power law (Pareto) distribution, being smaller than 10° in approximately half of the voxels. Given measurement inaccuracies, these values probably represent a close to ideal match between DT and fiber directions, confirming that (at high spatial MRI resolution) the diffusion tensor is a very accurate model of fiber orientation in about half of the analyzed tissue. Visual exploration (cf. Figure 3) as well as the correlation between unimodal histological FODs and the observed orientation match confirmed that, as expected, this was particularly the case in areas dominated by homogeneously oriented fiber pathways.

However, roughly one third of voxels showed an orientation mismatch between 10 and 45°. While angular differences of less than 10° are most likely caused by measurement inaccuracies, these higher values suggest additional sources of mismatch. We saw that in voxels containing crossing fibers, diffusion tensors tended to shift away from the primary toward the secondary histologically derived orientations. The amount of this shift was related to the prominence of the secondary orientations, as reflected by the correlation between angular differences and FOD parameters. Recently, there have been extensive considerations about the best approach to cope with this issue. One important way forward is to opt for more complex models based on high angular resolution diffusion imaging (HARDI), such as Q-Ball Imaging (Tuch, 2004), Diffusion Spectrum Imaging (DSI) (Wedeen et al., 2005), or Spherical Deconvolution (Tournier et al., 2004, 2007) in order to model the whole orientation profile within a voxel rather than only its principal direction. Likewise, a better spatial resolution should reduce the amount of voxels with problematic fiber crossings. The histological structure tensor approach produces fiber orientation distributions of high angular resolution at a “voxel size” which can be chosen arbitrarily. It would therefore be suited to investigate both the performance of complex dMRI models and the effect of different spatial resolutions on dMRI results in future studies.

There were also voxels which displayed angular differences of more than 45°. This cannot be accounted for by crossing fibers only, as such an averaging should give values of maximally 45°. Thus it can only occur when either the fiber material has no clear orientational structure or when DT and ST are based on different anatomical structures. In white matter, this was only observed in a very small proportion of voxels (3.3%). In gray matter however, this proportion was much larger (13.6%) and the distribution of angular differences showed a second peak at about 90°, indicating that in a sustantial subpopulation of voxels DT and ST orientations were orthogonal to each other. These voxels were found mostly at intermediate depths within gray matter, arranged in bands running parallel to the cortical surface (Figures 3A, 6C). In these bands, the histological orientations were tangential, caused by myelinated horizontal axons corresponding to the bands of Baillarger (Nieuwenhuys, 2013). The DT orientations in these regions, however, were oriented radially. The organized structure of pyramidal cell apical dendrites is a likely basis for these diffusion tensor orientations and should be invisible to the structure tensors due to the lack of myelin in dendrites. Such prominent radial bundles of dendrites can be found throughout gray matter and are most pronounced between layers III and V (Peters et al., 1997). In layer IV and Vb, these radial dendritic bundles cross the horizontally oriented bands of Baillarger which could explain the orthogonal orientation mismatch between DT and ST. This issue should be further elaborated in future studies using alternative histological labels, such as lipophilic dyes (Budde and Frank, 2012) or immunohistochemical staining of different neuronal or glial compounds. Such approaches could also enable the study of orientation distributions in layers I and II, which are weakly myelinated (Vogt and Vogt, 1919; Sanides, 1962; Nieuwenhuys, 2013).

Overall, these voxels with orthogonal DT-ST orientations were the main reason for the worse DT-ST match in gray than in white matter. Excluding them from the analysis, diffusion tensors were only marginally less aligned to stained fiber directions than in white matter. This points toward a good applicability of DTI not only to white matter but also at least to lower cortical layers revealing the characteristic radial structure of neurites here. This seems to run counter to the common experience with *in-vivo* diffusion MRI, but one has to keep in mind that the spatial resolution of our scan was much higher than what is currently used for *in-vivo* measurements. Therefore, an increase in diffusion MRI resolution to values significantly below 1 mm can considerably enlarge the scope of diffusion MR to include the analysis of gray matter structures. Due to the use of a myelin staining approach, we can at present not make predictions about DTI performance in the supragranular cortical layers. However, the fact that diffusion tensor orientations were much more variable in these layers than in infragranular layers suggests that DTI is still not a very well suited model for all cortical layers. This assumption is backed by recent studies on gray matter architecture using high resolution dMRI post-mortem, all of which employed dMRI models more complex than DTI (Bastiani et al., 2013; Kleinnijenhuis et al., 2013; Leuze et al., 2014).

### Scalar measures

We investigated the relation of FA, MD, and RD with white matter microstructure and gray matter myeloarchitecture. We found a group of medium to strong correlations between diffusion MRI measures and myelin histology measures: FA was positively linked with high staining intensity, a narrow peak in the histological FODs and a good agreement between the orientations of the diffusion tensor and the stained fibers. Conversely, high values of both mean diffusivity and radial diffusivity were linked to bimodal or diffuse histology FODs, weakly matching orientations, and a low staining intensity. These interrelations were strong enough to explain about 50% of the variance of FA, MD, and RD from histological variables in a multiple linear regression, which confirms and to some extent quantifies the role of myelin as an important modulator of diffusion anisotropy and overall diffusivity (Beaulieu, 2002).

Comparing the different correlation patterns of FA, MD, and RD, we found that FA was related most strongly to the orientational specificity of the FODs in the histological data whereas MD predominantly reflected staining intensity. This confirms that, as intended, FA is a measure of the anisotropy of fiber distributions, and MD of the overall amount of diffusion-restricting material (in this case myelin sheaths). RD showed a pattern somewhat midway between these extremes with a high correlation to both FOD characteristics and staining intensity.

However, the intensity of myelin stains does not only depend on the amount of myelin but also on the staining protocol and the achieved quality of the staining. First, the quality of myelin staining we were able to achieve was suboptimal compared to what can be obtained in a fresh sample, fixed, sectioned and stained soon after death. The tissue probe used in this study was fixed for about 1 year when it was stained, resulting in a paler stain than what is usually achieved immediately post-mortem. Even though unintended, for the purpose of identifying directions of individual fibers the relatively sparse staining proved helpful. Second, the Gallyas stain used here is known to label myelin well and produce clearly interpretable histology images but also to be less consistent than other protocols, i.e., it will often show a substantial amount of variance within and in between tissue probes. We have corrected for section to section variability by performing statistics sectionwise and pooling statistics over sections. However, the varying staining intensity within each section has to be seen as a possible noise source in our correlation results. These concerns for the interpretability of staining intensity apply much less to orientations because the structure tensor analysis is relatively robust to absolute image intensity and its global variations.

The analysis of orientation differences at different FA ranges revealed that FA had a high predictive value for the angular difference between ST and DT orientations. This gives FA some credibility as an indicator for the reliability of DTI results; in particular, it justifies its usage as a stopping criterion in tractography algorithms. However, FA must be evaluated differently for white and gray matter. In white matter, FA values above 0.3 seemed sufficient for a good ST-DT orientation match, whereas in gray matter this was already the case for values of 0.2 (within the confines discussed above). This was confirmed by investigating the white/gray matter boundary (Figure 6): at the transition from white to gray matter, a decrease of FA values was often observed although both diffusion tensors and the stained fibers still followed the same pathways. This could have potential practical implications for DTI tractography algorithms using FA thresholds as a stopping criterion. Our results indicate that, at high spatial resolutions, thresholds suitable for white matter tractography might be overly strict when applied to the deeper gray matter layers. Therefore, it could be advisable to use tractography algorithms in conjunction with a gray-white matter segmentation, adjusting FA thresholds across the gray-white matter boundary. In our post-mortem sample, 0.3 would be a suitable FA threshold for white matter and 0.2 for gray matter.

## Conclusion

DTI is one of the simplest mathematical models available for diffusion weighted imaging, yet by far the most commonly used. We verify quantitatively here that structures which are simple enough to be modeled by diffusion tensors (individual coherently aligned fibers) are indeed described very accurately. Hence when analyzing strong, preferably unidirectional fiber pathways, DTI still appears to be the best choice due to its short acquisition time and low sensitivity to noise which generally comes with simple models. Furthermore, FA, MD, and RD were confirmed as sensitive measures of microstructural tissue characteristics, particularly being related to density and dispersion of myelinated fibers. However, complex fiber architecture with more than one principal fiber orientation within individual voxels cannot be sufficiently modeled using DTI. We found that in such cases diffusion tensor orientations represent either a mixture of the existing fiber orientations for relatively low angle crossings (often occurring in WM) or a selection of one of the two directions (for the orthogonal crossings typical of certain gray matter layers).

We provide new insights into the neuroanatomical basis of diffusion MRI in gray matter, where it is recently applied more often as spatial resolution increases to the required submillimeter level. In the deeper cortical layers (V, VI), diffusion tensor orientations matched myeloarchitecture almost as well as in white matter, accurately modeling cortical afferent and efferent fibers. This implies that, at high spatial resolution, FA thresholds might have to be adapted in order to conduct tractography across the white/gray matter boundary. In layers V to III, diffusion tensors were mostly oriented radially toward the cortical surface reflecting unmyelinated structure, presumably bundles of apical dendrites, which are orthogonal to tangential myelinated fibers. Although DTI followed these structures robustly, this selectivity for a subset of oriented neuroanatomical structures indicates that multi-orientation models are more appropriate in superficial cortical layers.

In future studies, the approach of validating dMRI results with histologically obtained FODs can be extended into a number of directions. Due to its adjustable angular and spatial resolution, the structure tensor technique could be used to investigate complex dMRI models as well as the effect of different spatial resolutions on dMRI results. Furthermore, tissue clearing methods have gained some attention in recent years for their ability to create transparent blocks of brain tissue which are compatible with immunohistochemistry (Dodt et al., 2007; Chung and Deisseroth, 2013). With the appropriate protocols and ways to obtain digitized data sets, one could use these methods to derive 3D structure tensors from tissue probes. This would make dMRI-ST comparisons even more viable and open up new possibilities to histologically validate dMRI tractography.

## Funding

This work was supported by the European Research Council (ERC-2010-AdG) to AS and RG and Portugal's science funding agency (Ciência2007) to NL The funding sources were neither involved in the work on this study nor in the writing of this article.

### Conflict of interest statement

The authors declare that the research was conducted in the absence of any commercial or financial relationships that could be construed as a potential conflict of interest.
