# Supplementary material for: Histological validation of high-resolution DTI in human post mortem tissue
Source: Front Neuroanat. 2015 Jul 23;9:98. doi: 10.3389/fnana.2015.00098 (PMC4511840; doi:10.3389/fnana.2015.00098)
Supplement: Supplementary file 1 [file DataSheet1.DOCX]

**Fiber staining according to Gallyas (1979)**

Staining procedure for sections already mounted on coated (porcine gelatine) slides:

1. Rinse in destilled water
2. 30 min pyridine-acetic anhydride mixture (200 ml pyridine, 100 ml acetic anhydride)
3. 2 x 5 min destilled water
4. Ammonium silver nitrate solution (cover the solution) 45 min

0,3 g ammonium nitrate

0,3 g silver nitrate

In 300 ml destilled water

+ 0,9 ml 1 N NaOH (pH 7,5)

1. 3 x 10 min 0,5 % acetic acid
2. Physical developer (until sections turn brown, max. 3 min,

mix solution A 150 ml, solution B 75 ml und solution C 75 ml)

1. 1 min 1% acetic acid
2. 10 min 0,2 % potassium ferrocyanide
3. 1 min destilled water
4. 1 min 1% acetic acid
5. 2 x 3 min 0,5 % sodiumthiosulfate (3 g in 600 ml Aqua dest)
6. 3 x 4 min destilled water
7. Ascending sequence of ethanol (70%, 96%, 100%)
8. Rinse in xylole
9. Coverslip with Shandon xylene substitute mountant

**Stock solutions**

A: 50 g sodium carbonate in 1000 ml destilled water

B: 2 g ammonium nitrate

2 g silver nitrate

10 g tungstic acid in 1000 ml destilled water

C: 2 g ammonium nitrate

2 g silver nitrate

10 g tungstic acid

7,3 ml 35% formalin in 1000 ml destilled water

**Reference**

Gallyas F. 1979. Silver staining of myelin by means of physical development. Neurol Res. 1:203–9.
